# Effects of artificial honey and epigallocatechin-3-gallate on *streptococcus pyogenes*

**DOI:** 10.1186/s12866-022-02611-0

**Published:** 2022-08-26

**Authors:** Xiaoge Jiang, An Lin, Shijia Li, Yangyang Shi, Fangjie Zhou, Grace Gomez Felix Gomez, Richard L. Gregory, Chaoliang Zhang, Song Chen, Ruijie Huang

**Affiliations:** 1 State Key Laboratory of Oral Diseases, National Clinical Research Center for Oral Diseases, West China Hospital of Stomatology, Sichuan University, Chengdu, 610041 China; 2 Department of Orthodontics Dentistry, West China Hospital of Stomatology, Sichuan University, Chengdu, 610041 China; 3 Department of Pediatric Dentistry, West China Hospital of Stomatology, Sichuan University, Chengdu, 610041 China; 4 Department of Endodontic Dentistry, West China Hospital of Stomatology, Sichuan University, Chengdu, China; 5 Department of Oral Biology, School of Dentistry, Indiana University, Indianapolis, USA

**Keywords:** Pharyngitis, *Streptococcus pyogenes*, Artificial honey, Epigallocatechin-3-gallate, Natural derivatives

## Abstract

**Background:**

*Streptococcus pyogenes* is an important global human pathogen that causes pharyngitis, and antibacterial therapy has become an important part of the overall therapy for pharyngitis. As natural derivatives, honey and green tea are often recommended for patients with pharyngitis in traditional Chinese medicine without experimental theoretical basis on wether the combined effect of honey and green tea on pharyngitis is better than they alone. The aims of this study were to explore the effects of artificial honey (AH) and epigallocatechin-3-gallate (EGCG) on *S. pyogenes* and elucidate the possible mechanisms, which were investigated using MIC (the minimum inhibitory concentration), FIC (fractional inhibitory concentration) index, growth pattern, biofilm formation and RT-qPCR.

**Results:**

The MIC of AH on *S. pyogenes* was 12.5% (v/v) and the MIC of EGCG was 1250 μg/ml. The FIC index of AH and EGCG was 0.5. The planktonic cell growth, growth pattern and biofilm formation assays showed that AH and EGCG mixture had stronger inhibitory effect on *S. pyogenes* than they alone. RT-qPCR confirmed that the expression of *hasA* and *luxS* gene were inhibited by AH and EGCG mixture.

**Conclusions:**

AH and EGCG mixture can inhibit the planktonic cell growth, biofilm formation and some virulence genes expression of *S. pyogenes*, better than they alone. The combination of honey and green tea have the potential to treat pharyngitis as natural derivatives, avoiding drug resistance and double infection.

## Background

Pharyngitis is one of the most common diseases, of which etiology is complex, including pathogenic microorganisms, physical or chemical stimulation and so on. Streptococcal pharyngitis is an acute infection of nasopharynx and oropharynx caused by *S. pyogenes*, accounting for more than 37% of all diagnosed pharynx pain cases, up to 5%-10% in adults reports millions of cases each year worldwide [1, 2]. *S. pyogenes* is one of the main pathogenic bacteria causing pharyngitis, which can produce a variety of toxins, M protein, streptokinase, chain enzyme, hyaluronidase and other pathogenic factors [3–6]. Therefore, antibacterial therapy has become an important part of the overall therapy for pharyngitis. However, most of the current antibacterial therapies use a variety of antibiotics, and the widespread use of antibiotics easily leads to drug resistance and double infection [7, 8]. The emergence of antibiotic resistance among *S. pyogenes* and treatment failure has become an added concern globally, so it is of great interest to find natural derivatives with good antibacterial performance and tolerance that can replace antibiotic therapy [9, 10].

The antibacterial properties of honey have been recorded for a long time [11], but the specific mechanism is still unknown [12]. The factors that may play a bacteriostatic role include flavonoids [13], hydrogen peroxide [14, 15], low pH value and hyperosmotic environment formed by sugar [16]. In Maddocks’ study [17, 18], manuka honey is effective at inhibiting the development of biofilms, disrupting established biofilms of *S. pyogenes*, and inhibiting bacterial adhesion to the fibronectin, fibrinogen and collagen. Moussa et al. [19] found that the MIC (%) of honey types of Algeria to *S. pyogenes* ranged 25%-73%. Al-kafaween et al. [20, 21] found that Trigona honey and Malaysian Tualang honey were both effective inhibitors and virulence modulators of *Pseudomonas aeruginosa* and *S. pyogenes* via multiple molecular targets. Whereafter, the group of Al-kafaween [22] compared the antibacterial activity of Kelulut Madu honey (KMH), Jarrah honey (JH), Acacia honey (AH) and Gelam honey (GH) with that of Manuka honey (MH) and indicated that all honeys can inhibit *Pseudomonas aeruginosa* and *S. pyogenes* due to a decrease in expression of essential genes, suggesting that all honeys could potentially be used as an alternative therapeutic agent against certain microorganisms. At present, most studies about honey and *S. pyogenes* are based on wound infection rather than Streptococcal pharyngitis [23]. And studies have shown that both artificial honey and natural honey can inhibit bacterial proliferation and biofilm formation, but there are still differences between the two, and sugar is not the only determinant of honey's antibacterial effect [24].

Numerous studies have shown that tea polyphenols are one of the most promising natural antibacterial substances up to now, among which epigallocatechin-3-gallate (EGCG) is the most important component [25]. EGCG has significant antibacterial [26], antiviral [27], resisting arteriosclerosis [28], thrombosis [29], vascular proliferation resistance [30] and anti-tumor effect [31], and has become a research hotspot in recent years because of its antioxidant activity and anti-inflammatory properties [32]. Therefore, it is widely used in the treatment of various diseases. So far, there are a lot of studies about the bacteriostatic activity of EGCG or its derivative, such as growth-inhibition on *Staphylococcus aureus, Escherichia coli, Clostridium perfringens, Streptococcus mutans, Enterococcus faecalis, Porphyromonas gingivalis* and so on [33–36]. Sakanaka et al. [37] found that the inhibitory effect on the adherence of *P. gingivalis* onto the buccal epithelial cells is attributed to the presence of the galloyl moiety, which is ester-linked with the 3-OH of the catechin moiety in the polyphenolic compounds. Hull’s research [38] found that pretreating or post treating the kidney epithelial cells (RMKEC) with EGCG inhibited the attachment of the *S. pyogenes* to the cells in a dose dependent manner. The results indicated that EGCG can effectively reduce *S. pyogenes* cellular attachment and induce *S. pyogenes* cell death, which can be used as an adjunct to conventional antibiotic treatments.

Honey is a natural health care product containing a variety of nutrients and antibacterial effects. Green tea contains tea polyphenols which also have antibacterial and anti-inflammatory effects. The combination of honey and green tea can neutralize the bitter taste of tea, as well as achieve complementary effects. From ancient times to the present in China, honey and green tea combination is a popular drink and a way to treat pharyngitis. However, there is still lack of experimental theoretical basis and specific mechanism on wether the combined effect of honey and green tea on pharyngitis is better than they alone. It would be of great interest to explore the combined effect of honey and green tea on the viability, planktonic growth, biofilm formation, metabolism and the mechanism of its antibacterial action on *S. pyogenes*.

## Results

### The MIC and FIC index of AH and EGCG on *S. pyogenes*

**Fig. 1:**
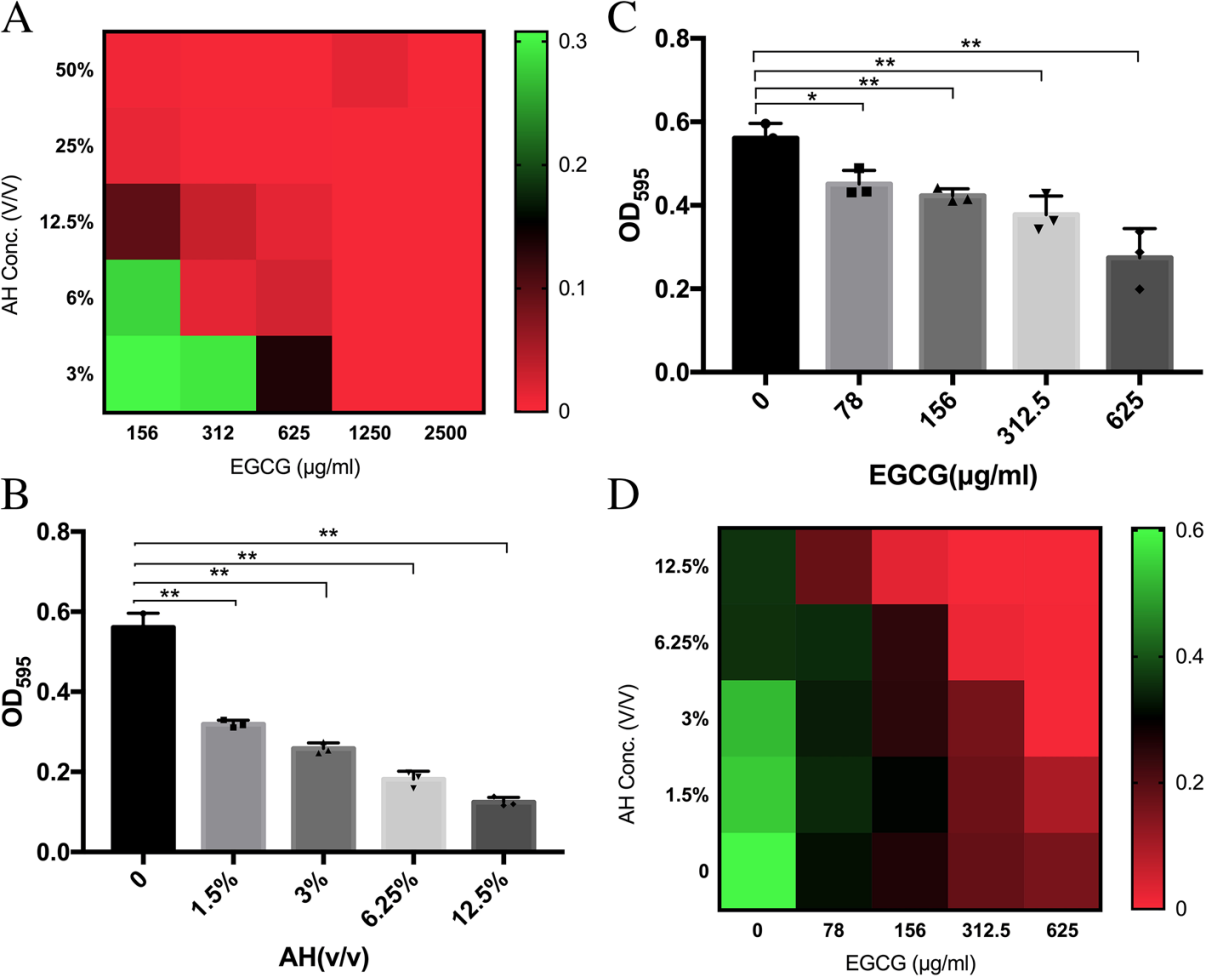
The FIC index of AH and EGCG on *S. pyogenes* (**A**). Effect of AH (**B**), EGCG (**C**), AH and EGCG mixture (**D**) on the planktonic cell growth of *S. pyogenes*. Data are expressed as mean SD. *P < 0.05; **P < 0.01

In this study, the MIC of AH on *S. pyogenes* was 25%. The MIC of EGCG on *S. pyogenes* was 1250 µg/ml. The FIC index of AH and EGCG combination on *S. pyogenes* was 0.5, so the combination of the two is a synergy effect (Fig. 1A).

### The planktonic cell growth of AH and EGCG mixture on *S. pyogenes*

The absorbance _(OD595)_ values represented the planktonic cell growth of the bacteria in varied conditions. As shown in the Fig. 1B, the 1.5%, 3%, 6.25% and 12.5% AH-treated groups had less biomass than the controlled (*P* < 0.05). With the increase of concentration, the inhibition of AH on the planktonic growth of *S. pyogenes* was more significant. In Fig. 1C, the 78 µg/ml, 156 µg/ml, 312 µg/ml and 625 µg/ml EGCG-treated groups had less biomass than the controlled group (*P* < 0.05), and the concentration-dependent inhibitory effect of EGCG on *S. pyogenes* could be clearly seen. The combined effect of AH and EGCG were showed in Fig. 1D. The experimental results demonstrated that 1) The OD_595_ value of *S. pyogenes* ranged from 0 to 0.6 when treated with different concentrations of AH and EGCG mixture. 2)When the concentration of artificial honey was fixed, with the increase of the concentration of EGCG, the absorbance _(OD595)_ values of *S. pyogenes* decreased gradually. 3)When the concentration of EGCG was fixed, with the increase of the concentration of AH, the absorbance _(OD595)_ values of *S. pyogenes* decreased. 4) The combined effect of EGCG and AH was stronger than they alone.

### Effect of AH, EGCG, and AH and EGCG mixture on the growth curve pattern

**Fig. 2:**
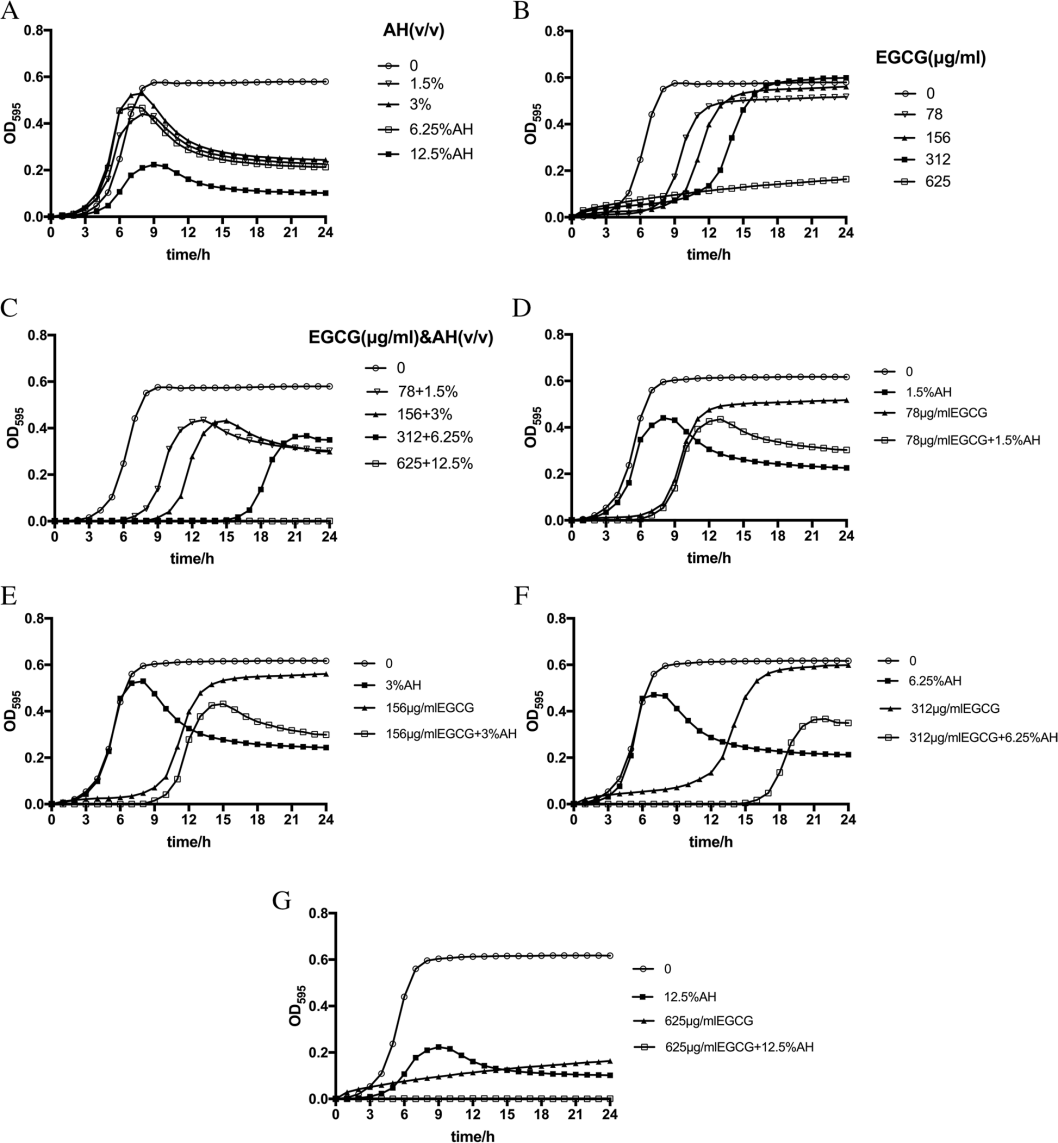
Effect of AH (**A**), EGCG (**B**) and AH and EGCG mixture (**C**) on the growth curves of *S. pyogenes*; effect of different concentration of AH and EGCG mixture on the growth curve of *S. pyogenes* coompared with AH and EGCG (**D-G)**

The growth curve (Fig. 2A) demonstrated that the absorbance of the plateau became lower with the increase of AH concentration, but the duration of the logarithmic growth phase had hardly changed. The growth curve (Fig. 2B) showed that with the increase of EGCG concentration, the duration of the logarithmic growth phase was prolonged, but the absorbance of the plateau did not change significantly except 625 µg/ml group. As shown in the Fig. 2C, when AH and EGCG mixture was added into the medium of *S. pyogenes*, the logarithmic growth period was prolonged and the absorbance of the platform was also decreased. According to Fig. 2D-F, the combined effect of EGCG and AH to prolong the duration of the logarithmic growth phase was stronger than EGCG, and the effect to decrease the absorbance was stronger than EGCG but weaker than AH. As shown in the Fig. 2G, 12.5% (v/v) AH combined with 625 μg/ml EGCG can inhibit the planktonic growth of *S. pyogenes* completely, stronger than they alone.

### Effect of AH, EGCG, and AH and EGCG mixture on the biofilm formation

**Fig. 3:**
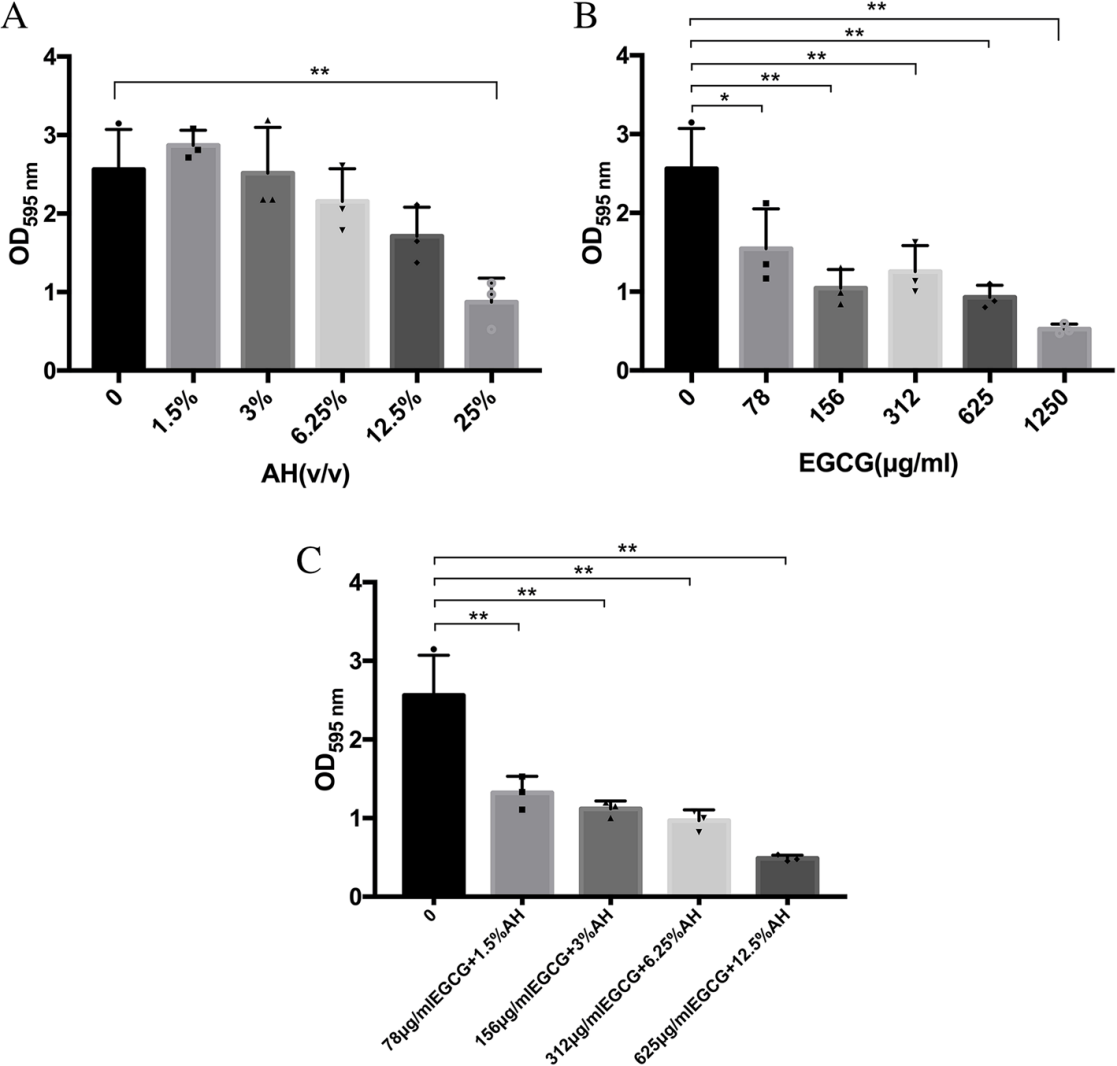
Effect of AH (**A**), EGCG (**B**) and AH and EGCG mixture (**C**) on the biofilm biomass of *S. pyogenes* analyzed by crystal violet assay. Data are expressed as mean SD. *P < 0.05; **P < 0.01

Crystal violet assay demonstrated that when the concentration of AH exceeds 25%, the biofilm volume decreased with the increase of AH concentration (Fig. 3A). AH in high concentration could inhibit the biofilm formation of *S. pyogenes*. According to Fig. 3B, EGCG can inhibit the growth of biofilms from low concentration (78 μg/ml). As shown in the Fig. 3C, all the four AH and EGCG-treated groups had significant inhibition effect on biofilm formation of *S. pyogenes*.

### Effects of AH, EGCG, and AH and EGCG mixture on the morphology of *S. pyogenes*

**Fig. 4:**
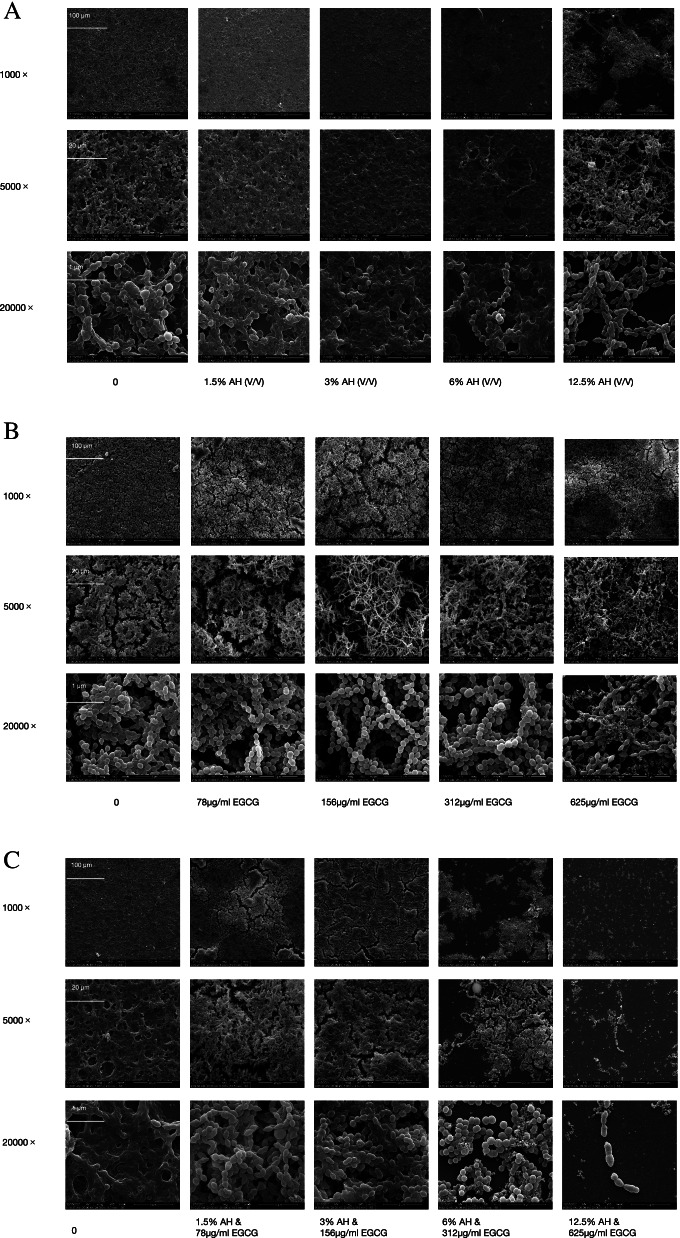
Effect of AH (**A**), EGCG (**B**) and AH and EGCG mixture (**C**) on the morphology of *S. pyogenes* by SEM observation of the biofilm after 24 h of incubation with the magnification of 1,000 × , 5,000 × , and 20,000 ×

Changes in density and structure of *S. pyogenes* were observed compared to control group after 24 h of treatment with AH at above four concentrations (Fig. 4A). In the absence of AH, clusters of *S. pyogenes* formed a uniformly distributed network structure. Low concentration of AH (1.5%, 3%) could increase the density of biofilm and stronger the network structure. But when the concentration of AH was high (6.25%, 12.5%), the density of biofilm decreased, and the network structure was damaged to varying degrees. As for the effect of EGCG, changes in density and chain-length of *S. pyogenes* could be seen compared to control group after 24 h of treatment with EGCG at these four concentrations (Fig. 4B). Compared with the control group, *S. pyogenes* had lower cell density at these four group. In the absence of EGCG, *S. pyogenes* had a higher cell density, shorter chain length and bacterial were more randomly distributed. As the concentration of EGCG increased (ranging from 78 to 312 μg/ml), bacteria aggregated together to form longer chains. When at the high concentration of EGCG (625 μg/ml), the chain gets short, but still longer than the control group. As shown in the Fig. 4C, 12.5% (v/v) AH combined with 625 μg/ml EGCG can inhibit the biofilm formation of *S. pyogenes* completely, stronger than they alone. Compared with the control group, the other four group of *S. pyogenes* had lower cell density, and the biofilm was also loose as the bacteria dispersed (Fig. 4C).

### Effects of AH, EGCG, and AH and EGCG mixture on gene expressions

**Fig. 5:**
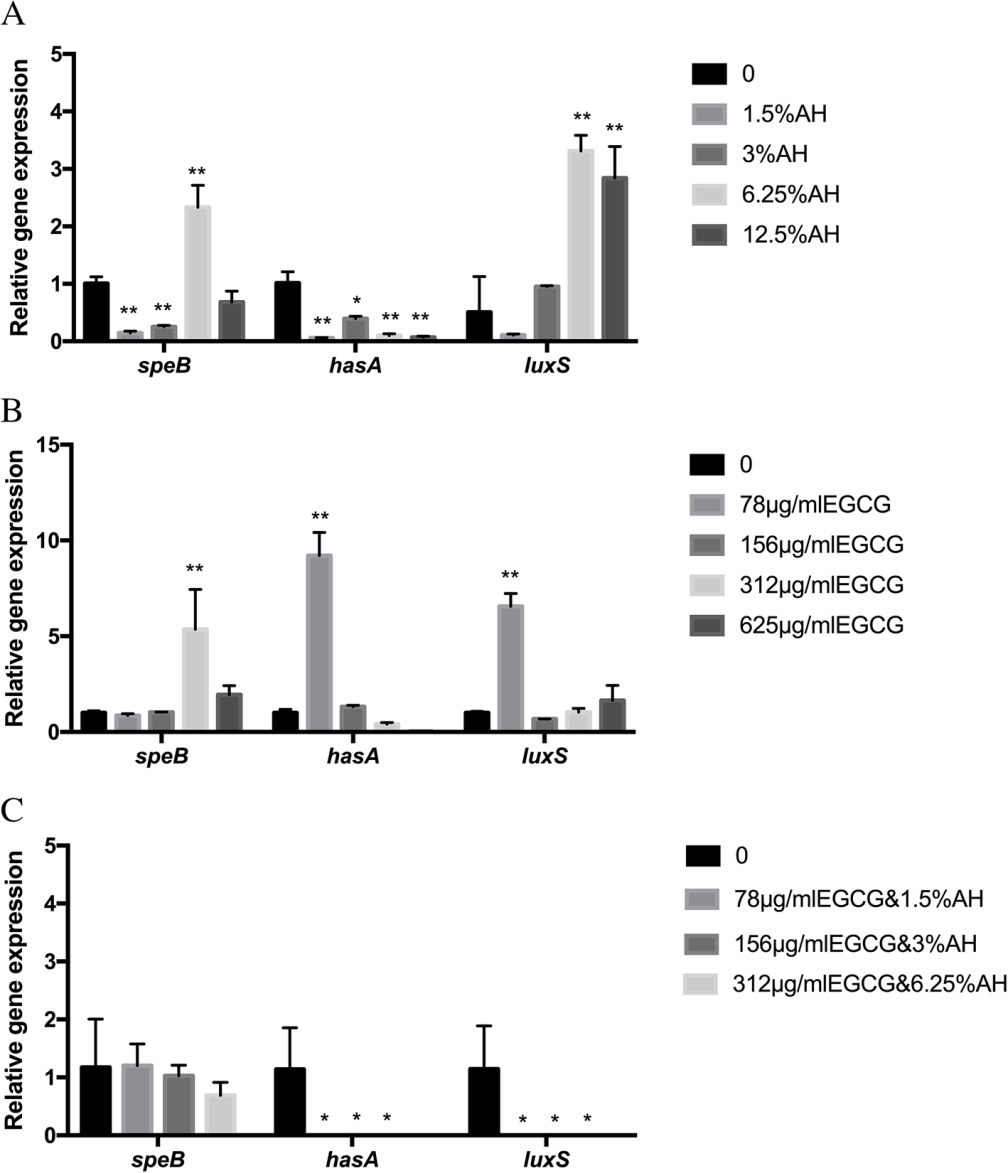
Expression of various virulence genes in *S.* pyogenes after treated with AH (**A**), EGCG (**B**) and AH and EGCG mixture (**C**) at sub-minimum inhibitory concentration (MIC) levels compared with control group. Gene expression was quantified by RT-qPCR with 16S ribosomal RNA as the internal control. Data are expressed as mean SD. *P < 0.05; **P < 0.01. *speB*, streptococcal pyrogenic exotoxins B; *hasA*, hyaluronan synthase; *luxS*, S-ribosylhomocysteine lyase

As is showed in the Fig. 5A, the expression of cysteine proteinase exotoxin (*speB*) was decreased at the low concentration of AH (1.5%, 3%) but increased significantly at high concentration of AH (6.25%). When at 12.5% concentration of AH, there showed no significant difference between the experimental group and the control (*P* > 0.05). Hyaluronan synthaseh (*hasA*) gene showed a trend to increase their expression at all the concentrations of AH except 3%. The expression of S-ribosylhomocysteine lyase (*luxS*) genes increased and exceeded that of the control group at high concentration of AH (6.25%, 12.5%). The effects of EGCG on virulence factors of *S. pyogenes* were analyzed by qPCR (Fig. 5B), and all these genes showed a trend to increase their expression at one of the concentrations of EGCG. When the concentration of EGCG was 312.5 μg/ml, the expression of *speB* genes increased and exceeded that of the control group (*P* < 0.01). When the concentration of EGCG was 625 μg/ml, the expression of *hasA* and *luxS* genes increased significantly. When the AH and EGCG mixture were added in the experiment groups, all the expression of genes was significantly decreased except *speB* (Fig. 5C).

## Discussion

*S. pyogenes*, the most pathogenic streptococcus, is the causative agent of many human soft tissue diseases, ranging from superficial (such as pharyngitis, pustular disease) to life-threatening diseases (such as cellulitis, necrotizing inflammation and myositis). There are many clinical treatment methods for *S. pyogenes* induced pharyngitis, in which the most common one is antibiotics. At present, there is a consensus among medical experts around the world on whether antibiotics should be used to treat acute pharyngitis. Once the bacterial infection is identified, penicillin should be preferred because *S. pyogenes* are sensitive to it [39]. When patients are allergic to penicillin, macrolides may be considered as an alternative. Cephalosporins may be considered in patients who are not highly sensitive to penicillin [40], and cephalosporins may be more effective than penicillin in cases of relapse [41]. Some scholars have also proposed that cephalosporins may be more effective than penicillin in treating streptococcal acute pharyngitis [42]. But the widespread use of antibiotics easily leads to drug resistance and double infection.

Honey and green tea are both the most promising natural derivatives with good antibacterial performance and tolerance right now. In traditional Chinese medicine, honey and green tea is often recommended for patients with pharyngitis. Studies have shown that both artificial honey and natural honey can inhibit bacterial proliferation and biofilm formation, but there are still differences between the two, and sugar is not the only determinant of honey's antibacterial effect. Green tea contains a variety of green tea catechins, and EGCG is the most important catechin found in green tea extract and also the most studied green tea extract among them. Due to the complex compositions and differences in manufacturers of honey and green tea, we just studied the effect of artificial honey and EGCG on *S. pyogenes*.

In order to investigate the antibacterial efficacy of AH or EGCG against *S. pyogenes*, the MIC of AH or EGCG was detected by the double dilution method in this study. AH or EGCG with different concentrations was co-incubated with bacteria, and the OD value of the bacteria in each concentration of AH or EGCG at 590 nm was measured by ultraviolet spectrophotometer. The MIC of AH on *S. pyogenes* was 12.5% and the MIC of EGCG was 1250 μg/ml. Synergy testing of AH combination with EGCG on *S. pyogenes* was performed by checkerboard method, and the interaction was determined according to calculated FIC index. The FIC index of AH and EGCG was 0.5, so the combination of the two is a synergy effect, which is usually thought of as advantageous. In the study of the effect of AH and EGCG mixture on the planktonic cell growth of *S. pyogenes*, it showed than when the concentration of AH was fixed, with the increase of the concentration of EGCG, the inhibitory effect of the mixture on the growth of planktonic bacteria was enhanced. And when the concentration of EGCG was fixed, with the increase of the concentration of AH, the inhibitory effect of the mixture on the growth of planktonic bacteria was enhanced. The combined effect of EGCG and AH was stronger than they alone. Due to the different concentration of artificial honey with different EGCG has a variety of results, 625 μg/ml EGCG and 12.5% (v/v) AH, 312 μg/ml EGCG and 6.25% (v/v) AH, 156 μg/ml EGCG and 3% (v/v) AH were chosen in the follow-up studies.

It is well known that the normal growth curve of bacteria in a suitable environment should pass through four periods: the delay period, logarithmic phase, stabilization period and the decay period. According to the results of the growth curve test, EGCG can prolong the duration of the logarithmic growth phase and lower its final amount at platform stage, and AH can decrease the absorbance of the platform significantly. The effect of AH and EGCG to prolong the duration of the logarithmic growth phase was stronger than EGCG, and the effect to decrease the absorbance was stronger than EGCG but weaker than AH. The biofilm, which is a multidimensional complex structure formed by bacteria, is considered an important pathogenic factor in many diseases. Our study further elucidated that EGCG can inhibit the growth of biofilm from low concentration (78 μg/ml), and AH in high concentration could inhibit the biofilm formation of *S. pyogenes*. All the four AH and EGCG-treated groups had significant inhibition effect on biofilm formation of *S. pyogenes*. All these indicated that AH and EGCG had stronger inhibitory effect on *S. pyogenes* than they alone.

*S. pyogenes* secretes a variety of proven or putative virulence factors which enable it to invade or otherwise affect human hosts in adverse manners. Among these protein toxins are a group of superantigens known as streptococcal pyrogenic exotoxins A, C, etc. (SpeA, SpeC) [43] and a cysteine protease known as streptococcal cysteine protease or SpeB [44]. The superantigens differs in disease isolates of *S. pyogenes.* However, the cysteine protease SpeB is present in all isolates of *S. pyogenes* and can be the predominant extracellular protein, accounting for up to 95% of total secreted protein [45–47], and is highly conserved in virtually all strains of the human bacterial pathogen *S. pyogenes* [48]. The active protease can decompose the extracellular matrix, complement components and immune globulin of the host cell and the surface adhesion factor, M protein and some other secreted proteins of *S. pyogenes*, destroy the defense system of the host, help the bacteria to escape from immune clearance [49]. The RT-qPCR results showed a significant increase in *SpeB* expression at high concentration of AH or EGCG. However, in the experiment of AH and EGCG-treated groups, the expression of *SpeB* had no difference with the control group, which means the combined effect of AH and EGCG can prevent *S. pyogenes* from enhancing its ability to resist host immune response in adverse environment.

Hyaluronic acid (HA) capsule plays an important part in invasiveness and virulence of *S. pyogenes* and is produced in the surface of the bacteria [50]. The capsule also acts as a shield to prevent *S. pyogenes* from oxygen damage by making oxygen diffusion much more slow through the capsule and isolating the cell from the environment [51]. In addition, the capsule is also necessary in the process of maturation into a tridimensional structure for biofilm formation [52]. The capsule is produced by the products of capsule operon which contains three genes, *hasA*, *hasB*, and *hasC*, respectively encoding hyaluronan synthase, UDP-glucose 6-dehydrogenase, and UDP-glucose pyrophosphorylase [53]*.* Among them, *hasA* codes for the enzyme responsible for the overall hyaluronic acid synthesis reaction, and is highly conserved among streptococcal strains [54]. The RT-qPCR results showed a significant decrease in *hasA* gene in AH and EGCG mixture groups. It can speculated that the combined effect of AH and EGCG can inhibit the produce of HA, so that stop the bacteria invasion and reduce the virulence. Meanwhile, the process of maturation into a tridimensional structure is prevent, which can explain the results of biofilm formation experiment and SEM experiment above. While AH or EGCG are not effective when used alone.

It has been demonstrated that bacteria can develop profitable actions for the community by means of interacting and establishing complex social behaviors with their siblings or other neighouring bacteria through a conserved chemical language [55]. Quorum Sensing (QS) is the process of communication where bacteria trigger specific phenotypical responses by producing, secreting and detecting chemical signals [56]. Genes regulated by QS are involved in behaviors across the population. These decisions are favourable when performed as a synchronized population instead of at the individual level, including biofilm formation, secretion of virulence factors, etc. [57]. The QS systems of *S. pyogenes* can be categorized into four groups: regulator gene of glucosyltransferase (Rgg), lantibiotic systems, Sil signaling system, and LuxS/AI-2 [58]. LuxS enzyme is required for the production of the autoinducer-2 molecule (AI-2). Since the *luxS* gene is present in the genomes of many Gram-negative and Gram-positive bacteria, AI-2 is considered to be a universal signal for cell-to-cell communication between species [59]. In *S. pyogenes*, *luxS* deletion leads to diminished activity of the *SpeB* protease and decreased production of HA capsule as a result of abnormal processing [60, 61]. The RT-qPCR results showed a significant decrease in *luxS* gene in AH and EGCG-treated groups, while in AH-treated or EGCG-treated groups, the *luxS* gene expression seemed to increased. It is speculated that when *S. pyogenes* is exposed to mild adverse events, the cell-to-cell communication increase, helping the bacteria resist adverse conditions. However, when the adverse factor is too much, the communication process is abnormal, which is adverse to *S. pyogenes.* The AH and EGCG mixture had the best effect to inhibit the expression of *luxS* and prevent the bacteria to develop profitable actions for the community and adapt to environmental stress.

## Conclusions

The AH and EGCG mixture can inhibit the planktonic growth, biofilm formation and some virulence genes expression of *S. pyogenes*, better than they alone. RT-qPCR confirmed that the expression of *hasA* and *luxS* gene were inhibited by AH and EGCG mixture, which can explain the reduction and destruction of biofilms. These results provided favorable evidence and possibility for the clinical use of AH and EGCG mixture. Compared with antibacterial therapy, the combination of honey and green tea may be suitable to treat or at least relief pharyngitis as natural derivatives. As a daily drink, the honey and green tea mixture is expected to reduce the occurrence of streptococcus pharyngitis. When targeting pharyngitis, honey and green tea mixture may have an adjuvant therapeutic effect on the relief of pharyngitis symptoms, thus reducing the use of antibiotics. The limitations of this study are as follows. There is still insufficient evidence on whether pharyngitis can be cured completely, and it is doubtful that drinking honey and green tea will have enough effect since the time of action on *S. pyogenes* in the pharynx is short. It can be considered to make honey and green tea mixture into lozenges and increase the frequency of use to prolong the exposure to bacteria. The obtained results only represent a phenomenon, and further studies should focus on the detailed mechanisms. Besides, animal models and the anti-inflammatory effect of honey and EGCG should also be investigated. Besides, only one strain of *S. pyogenes* was used in the present study, and considering the complexity of the genotypes, further studies to investigate the influence of AH and EGCG mixture among different emm genotypes pathogens should be carried out.

## Methods

### Bacterial strain and growth condition

*S. pyogenes* HSC5 strain was kindly provided by the State Key Laboratory of Oral Diseases (Sichuan University, Chengdu, China). Bacteria were streaked on blood agar plate before use. *S. pyogenes* was cultured in brain heart infusion broth (BHI; Oxoid, Basingstoke, UK) in an anaerobic environment (80% N_2_, 10% H_2_, and 10% CO_2_) at 37℃ for 24 h. Unless specified otherwise, the 24-h bacterial culture [1 × 10^7^ colony-forming units (CFUs) ml^−1^] in either BHI or BHIS [BHI plus 1% (wt vol^−1^) sucrose], to which twofold serial dilutions of the stock solution of EGCG were added in each experiment. The BHIS medium was used in the biofilm-formation assay. Absorbance was measured using Multiskan GO spectrophotometer (Thermo Fisher Scientific Company, USA).

### Reagent Preparation

Artificial honey (AH) was prepared as described by Wilkinson and Cavanagh [62]. It contains 40.5% fructose (Fisher Scientific Inc., Fair Lawn, New Jersey), 33.5% glucose (Sigma-Aldrich Co. LLC, Milwaukee, WI), 7.5% maltose (Fisher Scientific Inc.), 1.5% sucrose (Fisher Scientific Inc.) and 17% deionized water (w/w), and was filter sterilized through 0.22 μm syringe filter. EGCG (Solarbio, Beijing, China) was dissolved in dimethyl sulfoxide (DMSO; Sigma-Aldrich, St Louis, MO, USA) to a stock concentration of 12,500 μg ml^−1^.

### Minimum inhibitory concentration of AH and EGCG for *S. pyogenes*

To analyze the minimum inhibitory concentration (MIC) of AH for *S. pyogenes*, bacteria (1 × 10^7^ CFU/ml) were cultured in BHI mixed with different concentrations of AH ranged from 1.5% to 12.5% at 37℃for 24 h, then measured the optical density (OD) value at 595 nm using Multiskan GO spectrophotometer. To analyze the MIC of EGCG for *S. pyogenes*, bacteria (1 × 10^7^ CFU/ml) were cultured in BHI mixed with different concentrations of EGCG ranged from 78 to 1250 µg/ml at 37℃for 24 h, then measured the OD_595_ value using Multiskan GO spectrophotometer. The amount of bacteria in the medium was represented by the OD_595_ value, compared with the standard group. The MIC was defined as the lowest concentration of AH or EGCG that yielded a change in OD_595_ of ≤ 0.05 [21]. This experiment was independently repeated three times.

### Fractional inhibitory concentration index of AH and EGCG combination for *S. pyogenes*

To analyze the fractional inhibitory concentration (FIC) index of AH and EGCG mixture for *S. pyogenes*, 100% AH preparation was serially 1:1 (v/v) diluted in BHI, yielding 100%, 50%, 25%, 12.5%, 6.25%, 3%AH preparation, and 12,500 μg ml^−1^ stock concentration of EGCG was diluted in BHI yielding 5000, 2500, 1250, 625, 312, 156 μg ml^−1^. The solution of different concentrations of each pair of AH and EGCG combination was designed according to the checkerboard method, and 100 μl of each drug was added to each culture in per well in sterile 96-well-plates. *S. pyogenes* (1 × 10^7^ CFU/ml) were cultured at 37℃for 24 h in the 96-well-plates. The FIC of each agent was calculated as a ratio of the MIC when used in combination and the MIC when used alone. FIC index is the sum of the FIC of the two agents used in the combination [63]. FIC indexes were interpreted as previously defined synergy at a FIC index ≤ 0.5; additive at a FIC index > 0.5 to 1; indifference at a FIC index > 1- < 2; and antagonism at a FIC index ≥ 2 [64].

### Effect of AH and EGCG mixture on the planktonic cell growth of *S. pyogenes* by optical density value test

*S. pyogenes* (1 × 10^7^ CFU/ml) were cultured in BHI with different concentrations of AH (ranged from 0, 1.5%, 3%, 6.25%) and EGCG (0, 78, 156, 312, 625 µg ml^−1^) mixture (Table 1, experimentI), then incubated at 37℃for 24 h in sterile 96-well-plates (Fisher Scientific Inc.), 200μL culture per well. The turbidity of culture was read at OD_595nm_ using Multiskan GO spectrophotometer. The amount of bacteria in the medium is represented by the OD value, compared with the standard group. This experiment was independently repeated three times.

**Table 1:** the concentrations of AH and EGCG mixture used in current study

AH EGCG	625 µg/ml	312 µg/ml	156 µg/ml	78 µg/ml	0
12.5% (v/v)	experimentI experimentII experimentIII experimentIV	experimentI experimentII experimentIII experimentIV experimentV	experimentI experimentII experimentIII experimentIV experimentV	experimentI experimentII experimentIII experimentIV experimentV	experimentI experimentII experimentIII experimentIV experimentV
6.25% (v/v)	experimentI	experimentI experimentII experimentIII experimentIV experimentV	experimentI	experimentI	experimentI
3% (v/v)	experimentI	experimentI	experimentI experimentII experimentIII experimentIV experimentV	experimentI	experimentI
1.5% (v/v)	experimentI	experimentI	experimentI	experimentI experimentII experimentIII experimentIV experimentV	experimentI
0	experimentI experimentII experimentIII experimentIV experimentV	experimentI experimentII experimentIII experimentIV experimentV	experimentI experimentII experimentIII experimentIV experimentV	experimentI experimentII experimentIII experimentIV experimentV	experimentI experimentII experimentIII experimentIV experimentV

### Effect of AH, EGCG and AH and EGCG on the growth pattern of *S. pyogenes* by growth curve test

Growth curve assay of *S. pyogenes* was done by following the protocol of Al-Kafaween et al. with some modification [21]. *S. pyogenes* (1 × 10^7^ CFU/ml) were cultured in BHI with different concentrations of AH and/or EGCG (Table 1, experimentII) and seeded in sterile 96-well-plates (Fisher Scientific Inc.), 200 μl culture per well. The 96-well-plate was sealed by sterile mineral oil and inoculated in spectrophotometer (Spectra Max 190, Molecular Devices, Sunnyvale, CA) at 37℃. The turbidity of culture was read every hour during a 24 h period at optimal density equals to 595 nm (OD_595nm_). The absorbance value at each time point was calculated by subtracting the absorbance value of standard group from the raw absorbance value. This experiment was independently repeated three times.

### Effect of AH, EGCG and AH and EGCG mixture on the biofilm formation of *S. pyogenes*

Biofilm formation assay was done by following a protocol described by Al-Kafaween et al. with some modification [21]. *S. pyogenes* (1 × 10^7^ CFU/ml) was incubated in BHIS plus AH and/or EGCG at different concentrations (as described in Table 1, experiment III), in a 48-well microtiter plate for 24 h, in triplicate. BHIS with no bacteria was used as a negative control. The supernatant of each well was decanted, and the biofilm was washed twice with phosphate buffer saline (PBS), fixed with anhydrous methanol for 15 min, and stained with 0.1% (wt/vol) crystal violet (Sigma) for 5 min. After that, the residual dye was removed by distilled water. When the biofilm had dried slightly, 500 μl of absolute ethyl alcohol was added and the 48-well microtiter plate was shaken at room temperature (20 ℃) for 30 min. The absorbance of extracted crystal violet in absolute ethyl alcohol was measured at absorbance_595nm_. The absorbance_595nm_ value of the experimental group minus the negative control group represented the volume of the biofilm. This experiment was independently repeated three times.

### Effects of AH, EGCG and AH and EGCG mixture on the morphology of *S. pyogenes* biofilm by scanning electron microscopy (SEM)

The protocol of SEM assay was described by Al-Kafaween et al. [21] with some modification. The biofilm specimens of *S. pyogenes* (as described in Table 1, experiment IV**)** were grown on sterile glass slides at the bottom of 24-well microtiter plates for 24 h. For SEM, the biofilms were washed with PBS twice and then transferred to a new 24-well microtiter plate. 2.5% (v/v) glutaraldehyde was used to fix the biofilms overnight at 4℃. The biofilm samples were dehydrated using graded ethanol series (30%, 50%, 75%, 85%, 95%, and 100%; 15 min each). After dried overnight, samples were finally observed by SEM imaging (JSM-7500F, Japan) at magnifications of 1,000 × , 5,000 × , and 20,000 × . Each biofilm was scanned at three randomly selected spots.

### Effects of AH, EGCG and AH and EGCG mixture on the quantification of gene expressions of *S. pyogenes* by RT-qPCR

*S. pyogenes* was grown in BHIS in 6-well microtiter plates for 24 h (as described in Table 1, experiment V). Then bacteria in the biofilm were collected, resuspended in 250 µl of 20 mg/ml lysozyme (J&K Scientific), and incubated at 37 °C for 1.5 h to break the cell wall of *S. pyogenes*. After that, 800 µl of Trizol (Thermo Scientific) was added to each sample and placed at room temperature (20 °C) for 5 min. After adding 250 µl of chloroform, the mixture was blended by vortexing and placed at room temperature (20 °C) for 5 min. After centrifugation (12,000 g, 4 °C, 15 min), the stratification was obvious, and the clear liquid on the upper layer was collected and transferred into a sterile centrifuge tube. 500 µl of isopropanol (4 °C) was then added and the mixture was placed at room temperature for 10 min. The extracted RNA could be seen on the bottom of the centrifuge tube after centrifugation (12,000 g, 4 °C, 10 min). After discarding the supernatant, 75% ethyl alcohol was used to wash RNA and removed by centrifugation (7,500 g, 4 °C, 5 min). Finally, the extracted RNA was dissolved in DEPC water (Beyotime, Shanghai, China). The quantity and quality of the extracted RNA were measured and evaluated by a NanoDrop 2000 (Thermo Scientifific). cDNA was synthesized according to the instructions of HiScript III RT SuperMix for qPCR (+ gDNA wiper) (Vazyme, China). The PCR reagent (10 µl) contained 2 × ChamQ Universal SYBR qRT-PCR Master Mix (5 µl), cDNA sample (50 ng, 1 µl), forward and reverse primers (10 µM, 0.2 µl) and ddH_2_O (3.6 µl). RT-qPCR was performed on a CFX96 Real-Time System (C1000 Thermal Cycler; Bio-Rad, Hercules, CA, USA) using the recommended procedure. Specific primers of the virulence-related genes in *S. pyogenes* were listed in Table 2. A modified 2^−ΔΔ^ Ct method was used to caculated and determined the level of gene expression [21]. The experiment was performed triplicate.

**Table 2:** the specific primers of quantitative real-time PCR

Detected virulence factor	Primer sequence(5’ → 3’)	Reference
*speB*	F: TGACGCTAACGGTAAAGAAAACA R: GCCGCCACCAGTACCAAGAGC	Burton et al. (2006) [65]
*hasA*	F: GTGTGTTAGCACAGACCTATCC R: CTTCGAAAATAGTCCATAAGGC	Petros et al. (2009) [66]
*luxS*	F: ATGATAAAGTGTA R: TTAGATTACTGAG	Raja et al. (2014) [67]
*16S rRNA*	F: AAGAGTTTGATCCTGGCTCAG R: GGTTACCTTGTTACGACTT	Hassan et al. (2015) [68]

### Statistical analysis

Statistical analysis was performed using IBM SPSS Statistics for Windows (Version 20.0, Released 2011; IBM, Armonk, NY, USA). Data were expressed as mean ± SD. Differences among groups with different concentrations of AH, EGCG and AH and EGCG mixture and the untreated control group were evaluated using one-way ANOVA and Dunnett’s multiple comparisons test. A value of *P* < 0.05 was considered statistically significant.

## Data Availability

The data that support the findings of this study are available on request from the corresponding authors. In addition, the datasets supporting the conclusions of this article is included within the article and its additional file.

## Abbreviations

SD: Standard deviation
ANOVA: Analysis of variance
